# Age-Related Alteration of Risk Profile, Inflammatory Response, and Angiographic Findings in Patients with Acute Coronary Syndrome

**DOI:** 10.4137/cmc.s2118

**Published:** 2009-02-18

**Authors:** Hala Mahfouz Badran, Mohamed Fahmy Elnoamany, Tarek Salah Khalil, Mostafa Mohamed Ezz Eldin

**Affiliations:** Cardiology Department, Faculty of Medicine, Menoufiya University, Shebin El Kom, Egypt.

**Keywords:** age-Hs-CRP-risk profile, acute coronary syndrome

## Abstract

**Background:**

Coronary artery disease (CAD) is a major public health problem which in turn imposes a significant burden on health care systems because of high morbidity and mortality. Although the multifactorial etiology of CAD increases with age, but in recent years, the incidence is increasing among younger age groups.

**Objectives:**

In this study we aimed to evaluate the effect of age on risk profile, inflammatory response and the angiographic findings in patients with ACS.

**Patients and Methods:**

The study comprised 253 ACS patients. Seventy six (30%) with UA, 56 (22%) with NSTEMI and 121(48%) with STEMI diagnosis. The value of Hs-CRP, lipid profile, cardiac enzymes, risk factors, EF% and angiographic score were analyzed and compared in different age groups.

**Results:**

Group 1 (n = 68) with age <45 years, group II (n = 110) with age ≥45–<65 years and group III (n = 75) ≥65 years. Group I had more prevalence of male sex, smoking, family history, hypertriglyceridemia and low levels of HDL (P < 0.01), higher incidence of STEMI (P < 0.01) and lower prevalence of UA (P < 0.01). Diabetes mellitus, hypertension, and female gender were more common in older groups. Hs-CRP was significantly lower in the young age (group I). Group I showed a preponderance of single-vessel disease, lower coronary atherosclerotic score and prevalent left anterior descending artery (LAD) involvement compared with older age groups. Hs-CRP was positively correlated to severity of CAD only in older groups. Stepwise multiple regression analysis showed that age, male gender, cardiac enzymes and EF% were common predictors of multivessel disease. Smoking was independent predictor in young patients <45 years while diabetes and Hs-CRP was the key predictor in older patient groups.

**Conclusion:**

Young patients with ACS had different clinical, angiographic and biochemical profile. Hs-CRP peak concentration did not correlate with angiographic findings in young patients that could be attributed to different risk profile and discrete underlying mechanism.

## Introduction

Acute coronary syndromes (ACS) are a major health problem and account for a large proportion of the total number of hospitalizations all over the world.^1^ There is a general agreement on a multifactorial etiology of coronary artery disease (CAD) and that the incidence of disease increases with age.^2^ Nevertheless, it has been recognized in young age groups more frequently in recent years.^3^–^4^ Worldwide, about 4% of patients with myocardial infarction (MI) are younger than 40 years of age.^5^ These patients frequently have characteristics that are different from those seen in older patients. While conventional risk factors clearly play a major role in the predisposition to MI, a significant number of young patients with ACS do not have any of the conventional risk factors, except for a family history of CAD.^6^ Furthermore, some previous studies have shown a correlation between C-reactive protein (CRP) and the presence of atherosclerosis,^7^ whereas others have not found a correlation.^8^ CRP might lose much if not all of its predictive value in both secondary and primary risk settings after adjustment for quantitative angiographic measures of CAD. On the other hand, CRP might add independently to measures of CAD extent and severity. While there are some studies from developed countries on the coronary angiographic profile and risk factor analysis of CAD patients at all ages,^9^,^10^ there are not enough published data addressing this problem in acute setting of CAD.

## Aim of the Work

To investigate the effect of age by itself on coronary risk factors, inflammatory response and angiographic findings in patients with ACS.

## Patients and Methods

Patients admitted to the coronary care unit (CCU) due to unstable angina pectoris or non-ST elevation acute myocardial infarction (NSTEMI) or ST elevation acute myocardial infarction (STEMI) from December 2006 to January 2008 were considered candidates for the study.

### Inclusion criteria

Inclusion criteria were defined as onset of chest discomfort in the prior 48 hours in patients with positive troponin (troponin I ≥ 1.0 ng/ml or troponin T ≥ 0.1 ng/ml) and/or electrocardiographic changes consisting of transient ST-segment depression (≥0.05mV) or T wave inversion (≥0.1 mV). Positive troponin patients had their index event defined as acute myocardial infarction and the remaining subjects were labeled as unstable angina.^11^ Acute STEMI was defined as chest pain lasting for >30 min, characteristic ST-segment elevation of ≥0.1 mV in two or more contiguous leads on ECG, and a creatine kinase (CK)&CK-MB values more than twice that of the highest reference laboratory value.^12^

#### Exclusion criteria

Patients with previous left bundle-branch block, pacemaker rhythm, acute inflammatory illness (within the last month), history of malignancy or secondary conditions that could precipitate angina (anemia, arrhythmias, fever) were not included in this study. Patients with cardiac valve disease, acute or chronic liver disease, and infectious diseases were also excluded from the study. None of the patients included in the study had ongoing systemic or cardiac inflammatory processes. All patients gave written informed consent before study entry and the study was approved by the local research ethics committee.

#### Patient selection

We studied 253 consecutive patients [age ranged from (32–85 years), 76.6% men] admitted to CCU unit for the assessment of angina chest pain. Seventy six (30%) patients had UA; fifty six (~22%) ACS patients had NSTEMI and 121 (~48%) had STEMI. The clinical management of ACS and the decision to proceed to angiography was left to the discretion of the managing cardiologist who was unaware of results regarding inflammatory markers. All enrolled ACS patients underwent coronary angiography during hospital admission or within three weeks after discharge.

#### A-Clinical characterization

Patient’s baseline clinical characteristics were evaluated. Clinical profiles included age, sex, smoking history, hypertension, diabetes mellitus (DM), previous history of CAD and family history of CAD. Smoking was classified as smoker and non-smoker. A smoker was defined as a patient who had smoked cigarettes regularly at least 1 cigarette/day within 2 years before study inclusion. A non-smoker was defined as a patient without smoking a cigarette in his or her life or quit smoking at least two years before inclusion in the study. Patients were diagnosed as hypertensive if told by a physician that they had hypertension or if they were using antihypertensive medication or if either systolic blood pressure ≥140 mm Hg or diastolic blood pressure ≥90 mm Hg was detected at least twice during admission. DM was diagnosed as a fasting blood glucose ≥126 mg/dl at least two times in admission or if they were treated with hypoglycemic agents or insulin.^13^ Family history of CAD was positive if at least one of the parents, siblings or children had manifestations of cardiovascular disease before the age of 55 years in males and 65 years in females. Body mass index (BMI) was calculated using the following formula: weight (kg)/height^2^ (m^2^).^13^ Dyslipidemia is diagnosed if plasma lipid analysis shows one or more of the following: hypercholesterolemia [total cholesterol (TC) ≥200 mg/dl and/or low-density lipoprotein (LDL-C) ≥130 mg/dl]; hypertriglyceridemia [triglycerides (TG) ≥150 mg/dl] and low levels of high-density lipoprotein (HDL-C) [HDL-C < 40 mg/dl in males and HDL-C < 50 mg/dl in female].^14^

##### B-Echocardiographic examination

After patient stabilization and within days of the onset of symptoms, the patients were subjected to detailed echocardiographic examination for assessment of LV function and wall motion abnormalities. Conventional M-mode, two-dimensional, pulsed Doppler and color flow Doppler echocardiography were performed using the commercially available **ACUSON 128 XP 10 C**. From apical and parasternal views visual assessment of regional wall motion was performed. Septal and posterior wall thickness during diastole was measured. End systolic diameter and volume (ESD and ESV), end diastolic diameter and volume (EDD and EDV), fractional shortening and ejection fraction by Simpson’s formula were measured.

#### C-Laboratory assays

In addition to routine laboratory work-up including, blood urea and serum creatinine, fasting and 2 hour post-prandial blood sugar, TC, TG, LDL-C and HDL-C patients were subjected to laboratory analysis of cardiac enzymes, CK-MB, total CPK, LDH. A blood sample to measure high sensitivity C reactive protein (Hs-CRP), as an inflammatory marker, was drawn immediately at hospital arrival, targeting the least possible time delay from symptoms onset before any therapeutic interference.

#### High sensitivity C-reactive protein

Fasting blood samples were obtained from ACS patients at the time of admission to CCU. Hs-CRP was measured using a commercially available immune-enzymatic method (Dade-Behring, Newark, Delaware, U.S.A.) with a precision limit of 0.3 mg/L and variation coefficient of 7.6%.^15^ Analytical precision of the Hs-CRP-Latex assay was 7.6% at a level of 1.02 mg/L, 3.3% at 1.79 mg/L and 1.3% at a level of 4.36 mg/L. Samples outside the analytical range of the high sensitivity CRP-Latex assay were analyzed by the CRP-Latex assay in the normal application.^15^

#### Angiographic analyses

Diagnostic coronary angiography (CA) was carried out in all patients using Judkins technique. Quantitative analysis of coronary arteries was performed with the computer-assisted Coronary Angiography Analysis System. End-diastolic frames from each arteriogram were selected for analysis.^16^ The percentage diameter stenosis (DS) was assessed in different projections and the highest value of each lesion, was chosen. Images of the coronary tree were obtained with the digital **SIEMENS HICOR 1000** set system. Two experienced cardiologists who had no knowledge of the patients’ clinical characteristics and biochemical results reviewed all angiographic images to assess the extent of CAD and morphology of all coronary artery stenoses.

##### Coronary angiograms were scored according to two scores

1. *Vessel score:* This was the number of vessels with a significant stenosis (50% or greater reduction in lumen diameter). Degree of stenosis was defined as the greatest percentage reduction of luminal diameter in any view compared with the nearest normal segment and was determined visually. Scores ranged from 0 to 3, depending on the vessels involved. Left main artery stenosis was scored as single vessel disease.^17^

2. *Severity score*: Using the scoring system described by Reardon et al.^18^ for analysis, the coronary circulation was divided into eight proximal segments. Disease in the distal segments was not considered because of difficulty in quantitating the severity of lesions in these areas. The eight proximal segments scored included the left main coronary artery, the left anterior descending artery (LAD) up to the junction of the middle and distal third of the vessel, the proximal third of the major septal branch of the LAD, the proximal third of the major diagonal branch of the LAD, the left circumflex coronary artery (LCX) up to the junction of the middle and distal thirds of the vessel, the proximal third of the major obtuse marginal branch of the LCX, the right coronary artery (RCA) up to and including the origin of the posterior descending coronary artery (PDA), and the proximal third of the PDA. In cases in which the PDA was supplied by the LCX vessel (LCX dominance), lesions in the LCX up to the origin of the PDA were included, as were lesions of the RCA up to the origin of the middle and distal thirds of the vessel. The PDA was scored identically for RCA and LCX dominant circulations. The percentage by which each lesion in the proximal coronary circulation narrowed the artery was assessed according to the maximal narrowing of the diameter of the artery in all projections. The severity of the proximal coronary disease was assessed by assigning points to each lesion as follows: less than 50% stenosis of the luminal diameter, 1 point; 50% to 74% stenosis, 2 points; 75% to 99% stenosis, 3 points; total obstruction, 4 points. The points for each lesion in the proximal coronary circulation were summed and a score for severity of coronary atherosclerosis was obtained. In previous study, the coefficient of variation between two angiograms analyzed several months apart without knowledge of the previous score was 4.9%.^18^

#### Statistical analysis

The collected data were tabulated and analyzed by SPSS statistical package version II on IBM compatible computer. Quantitative data were expressed as mean value ± SD and analyzed with student t-test for comparison of normally distributed values, and Mann Whitney U test for abnormally distributed ones. Chi-square test was used for analysis of qualitative data. Spearman correlation coefficient test was used to detect the association between 2 quantitative variables. A value of P < 0.05 (test of significance at 5% level) was considered statistically significant. Pearson correlation coefficient was used to evaluate the relation of different clinical, echocardiographic and angiographic variables to Hs-CRP and the relation of these variables to coronary artery scoring. Multivariate analysis was used to assess the predictors of multivessel disease in ACS patients.

## Results

### Patient population and demographics

The present study included 253 patients (194 males, 59 females) with ACS with age ranged from (32–85 years) recruited from CCU. To examine the relationship of age to risk profile, Hs-CRP and the severity of CAD in patients with ACS, patients was classified according to their age into 3 groups:

- *Group I:* included 68 patients with age <45 years.
- *Group II:* included 110 patients with age ≥45–<65 years.
- *Group III:* included 75 patients with age ≥65 years old.

Baseline demographics are presented by age category in table (1). Young ACS patients <45 years old represented 26.9% of total population. While older group ≥65 years represented 29.6% of total population. The residual 43.5% of ACS were in middle age between 45–65 years age.

**Figure 1.**
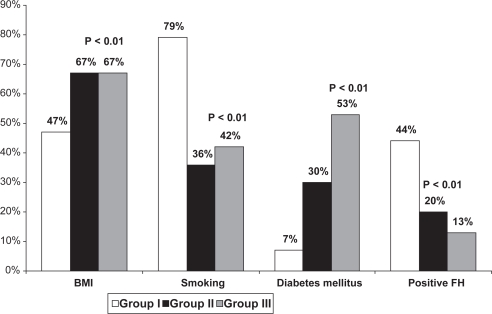
Prevalence of risk factors in studied patients with ACS.

**Figure 2.**
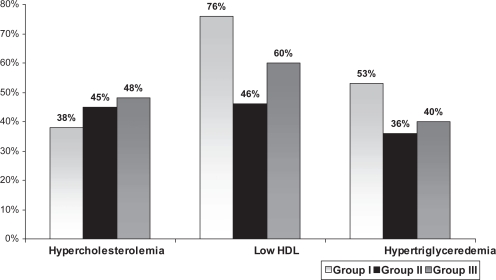
Comparison of dyslipidemia between different age groups (P < 0.01).

Male sex, smoking, positive family history, hypertriglyceridemia and low levels of HDL were more prevalent in group I while DM, hypertension and female gender were more common in group II and III. (Table 1, Figs. 1 and 2). ACS patients ≥65 years (group III) had higher incidence of diabetes compared with group II (P < 0.001) and lower HDL (P < 0.01) that could be related to physical inactivity. There was statistically significant difference in obesity prevalence between the groups. Group II and III had higher prevalence of obesity compared to young group (P < 0.01).

### Clinical presentation and medications of study patients with ACS

From total of 253 subjects enrolled who had angiographic and baseline demographic information and a baseline Hs-CRP level, patients presenting with symptoms consistent with unstable angina (UA) 76 comprised (~30%) of the study cohort; NSTEMI (n = 56) presentation made up ~22%; while STEMI (n = 121) comprised the remaining 48%. As regards to SBP and DBP, group II and III had higher levels in comparison to group I (144 ± 26 and 150 ± 35 vs. 122 ± 11 mmHg for SBP, 89 ± 23 and 94 ± 19 vs. 74 ± 10 mmHg for DBP, P < 0.01 for each respectively) where hypertension was more prevalent in such groups (group II and III).

**Figure 3.**
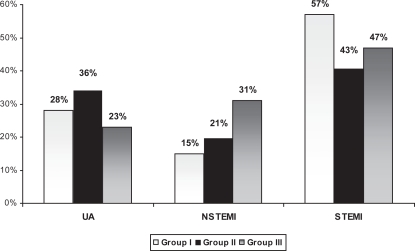
Comparison of clinical presentation with ACS in different age groups (P < 0.01).

Comparing the three age groups in their clinical presentation, young ACS patients (group I) had higher incidence of STEMI compared with group II and III [39(57%) vs. 47(43%) and 35(47%) P < 0.01] and lower prevalence of UA [19(28%) vs. 40(36%) and 17(23%) P < 0.01] respectively. Additionally, older group had higher prevalence of NSTEMI in comparison to group I [23(31%) vs. 10(15%) P < 0.001] and group II [23(31%) vs. 23(21%) P < 0.01] respectively (Fig. 3).

Rates of medications during hospitalization were 87.7% for aspirin, 86.9% for nitrate, 73.5% for angiotensin converting enzyme (ACE) inhibitors or ARBs, 71.1% for beta-blockers and 82.2% for statins, 89.3% for clopidogrel. No significant difference between different age groups in prescribed medications during hospitalization. Thrombolytic therapy was started more frequent in younger age group [32(47%) vs. 40(36%) and 29(39%) P < 0.01].

Among the entire group, angioplasty was recommended for 21 patients (8.3%), 24 patients (9.4%) were sent for surgery and medical therapy was prescribed to the 208 patients (~82%).

### Laboratory findings in studied groups: (Table 2)

Plasma glucose levels of group III patients in our study population were commonly not under control. Comparison of the fasting glucose, TC, HDL, LDL and TG levels of the patients are shown in Table 2. TC were significantly increased in older age groups (≥45 years) compared with group I (P < 0.001), LDL-C and HDL-C were significantly higher, P < 0.001, P < 0.01 in group II and III for LDL-C and P < 0.01 for HDL-C while TG was significantly lower (P < 0.001, P < 0.01) respectively compared with group I.

Cardiac enzymes (CPK, CK-MB and LDH) in patients with STEMI were significantly elevated in group II and III compared with younger group despite higher prevalence of STEMI in this group. This could be ascribed to greater number of the other two groups and larger number of diseased coronaries and infarct areas.

**Figure 4.**
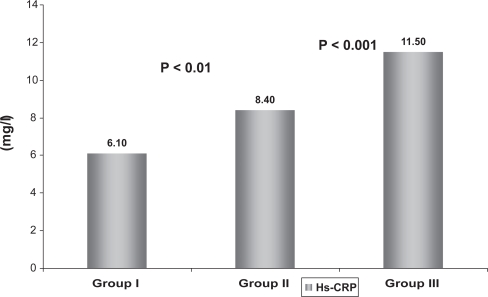
Comparison of Hs-CRP between different age groups.

Besides, Hs-CRP was significantly lower in younger age group (group I) in comparison to group II and group III (P < 0.01, P < 0.001, respectively) and the higher values were observed in older age group (group III) (P < 0.01 versus group II). Serum creatinine and blood urea were significantly higher in older age group (P < 0.01) (Fig. 4).

### Echocardiographic findings in studied populations

From all echocardiographic measurements merely EF% was significantly lower in group II (52.8 ± 13.1%) and III (49 ± 5.8%) compared with younger group I (58.3 ± 6.1%), (P < 0.01). Also, wall motion abnormality was significantly prevalent in older groups [group II: 74(67%) and group III: 55 (73%) vs. group I: 20 (29%), P < 0.001] respectively.

### Analysis of angiographic data in ACS patients

**Figure 5.**
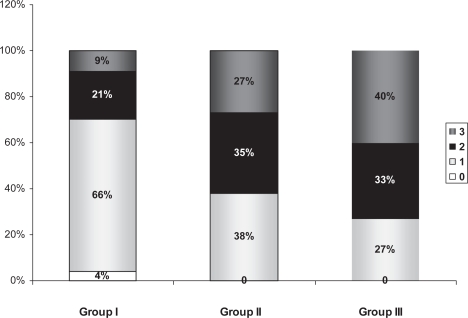
Comparison of vessel score in the different age groups.

**Figure 6.**
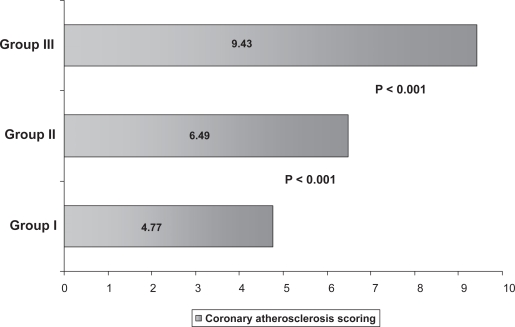
Severity scoring of CAD in different age groups.

Three patients (4.0%) of group I had normal coronary arteries (vessel score 0) which is not seen in any of the older patient groups. Besides, multivessel disease was detected more frequently in older patients (group II, III vs. group I) (62% and 73% vs. 29%, p < 0.001). Patients in group I showed a preponderance of single-vessel disease compared with patients in group II and III (p < 0.001) and significantly less common in group III compared with group II (P < 0.01) (Fig. 5). The prevalence of critical LAD involvement was different between the studied groups. Group I and III had prevalent involvement of LAD compared with group II (P < 0.01). However RCA and LCX significant stenoses were more common in group II compared with group I (P < 0.01) and group III (P < 0.01). Coronary artery score was significantly lower in younger group (4.77 ± 2.50) in comparison to group II and III (6.49 ± 6.17, 9.43 ± 6.61) P < 0.01, P < 0.001 respectively. (Fig. 6) There was no statistically significant difference between groups in the frequency of left main coronary artery stenosis. Coronary angiographic findings between the two groups are shown in Table 3.

### Correlations of CRP with risk factors of CAD: (Table 4)

Despite no relation was detected between prevalence of risk factors and plasma level of Hs-CRP, increasing age of patients was associated with higher values of Hs-CRP during acute coronary settings. Also Hs-CRP was positively correlated with the cardiac enzymes (CPK: r = 0.337, P < 0.001) and (LDH, r =0.319, P < 0.007), triglycerides level (r = 137, P < 0.03) and negatively with EF% (r = −0.265, P < 0.002) in the entire cohort of ACS patients. As regard the severity of coronary diseases, Hs-CRP was positively correlated with coronary severity score (r = 0.249, P < 0.002) and the number of vessels diseased (r = 0.314, P < 0.001) just in whole ACS patients.

### Relationship of Hs-CRP to severity of CAD in different age groups

When taken individually, Hs-CRP was positively correlated to severity of coronary diseases in the middle age group (group II, P < 0.03) and the old age group (group III, P < 0.01) but not in the young group <45 years. However, age, which was also correlated with Hs-CRP, showed positive correlation to severity of coronary disease in all age groups (P < 0.01, 0.02, 0.001 respectively).

Male gender was associated positively with more severe CAD. While smoking was positively correlated to the extent of CAD in young patients with ACS (P < 0.001). Diabetes mellitus was related significantly to CAD severity in patients ≥45 years (P < 0.001, 0.009 respectively). The univariate analysis revealed that cardiac enzymes also related to the extent of CAD while left ventricular EF% was negatively correlated with it P < 0.01 (group I), P < 0.02 (group II), P < 0.01 (group III), (Table 4).

After stepwise multiple regression analysis, independent predictors of the multivessels disease were different according to age group. While age, male sex, cardiac enzymes and EF% were common predictors of multivessel disease in all age groups, smoking was independent predictor in young patients <45 years old (OR 121, 95% CI: 0.023–0.226, P < 0.001) while diabetes and Hs-CRP were the key predictors in older patient groups (group II, III) (OR 0.167, 95% CI: 0.12–0.33, P < 0.0001, OR 2.01, 95% CI: 1.14–3.23, P < 0.007) respectively. (Tables 5, 6).

## Discussion

Premature CAD has been defined as having an age of onset ranging from 30 to 56 years in various studies. We selected an age cut-off point of 45 years to define a premature coronary artery disease based on previous epidemiologic studies.^19^ In this study, when comparing traditional risk factors for CAD, cigarette smoking was the most prevalent risk factor, with 79% of ACS subjects being smokers. This was followed by dyslipidemia; 76% had low HDL, 53% of patients had hypertriglyceridemia. This is followed by the prevalence of positive family history (44%). There was a statistically significant difference in low HDL cholesterol levels and triglycerides in young as compared with older ACS patients.

Our findings that cigarette smoking, positive family history and low HDL are the most common risk factors in younger patients with ACS, while hypertension and diabetes mellitus are more common in older ones, are in agreement with earlier observations.^9^,^14^,^20^,^21^ Smoking and low level of HDL were the main predictors for multivessel disease in premature ACS while diabetes and Hs-CRP were the most common risk predictors among older patients in our study.

A strong familial tendency was seen in our young ACS population; these observations concur with findings in a South American study in which the prevalence of a positive family history was 31% for MI cases compared with 15% in control subjects^22^ and support the presumption of the role of underlying genetic mechanisms.

Several studies have identified genetic predisposition in an estimated frequency between 5 and 14% in white Caucasian populations^23^,^24^,^25^ and is considered by some to be a genetic risk factor for MI.^23^,^26^ This association may be particularly evident in those with early onset (<45 years of age) CAD.^27^

Furthermore, in the existing study, the prevalence of diabetes was low in our young patients (7%) which are in agreement when compared with the findings of Imazio et al.^28^ in Caucasians; where they described diabetes as being more prevalent in an older population. The relatively low incidence of hypertension (23%) in our study is similar to the results reported by Seedat and Mayet,^29^ who found a prevalence of hypertension in 19% of their Indian-based population. These findings suggest that neither diabetes nor hypertension are major risk factors for ACS in young patients.

In the present study, Hs-CRP was considerably elevated in older age groups compared with young patients with ACS. This could be explained by different risk profile especially the prevalence of diabetes. Sanchez et al.^30^ ascertained that inflammation appears more evident in diabetic than in nondiabetic patients with ACS as assessed with CRP concentration and other inflammatory markers.

Besides, in the current study, we have shown that in ACS, Hs-CRP significantly higher and correlates with the extent of vascular disease in middle age and elderly patients. Also the independent contribution of Hs-CRP to risk assessment remains very large. However, the degree of this association in young patients with ACS is non considerable.

Because Hs-CRP is not correlated with angiographic scores in young group, it appears that Hs-CRP is stimulated not only by the extent of atherosclerosis but, importantly, by other factors. It is postulated that Hs-CRP is a measure of inflammed, unstable atherosclerotic plaque (both angiographically visible and occult), whereas angiography indicates only the extent of visible plaques. Hs-CRP also may reflect systemic but nonvascular chronic inflammatory and infectious processes that impact risk of acute coronary events by influencing levels of circulating proinflammatory and prothrombotic factors.

Additionally, the present study indicates that young patients <45 years have a higher frequency of angiographically less extensive disease and single vessel disease compared with other groups. Similar results have been reported by other authors.^31^–^33^ Histopathologic studies have demonstrated that atherosclerotic plaques in young patients with coronary artery disease are characterized by a large amount of lipid-containing foam cells and relative lack of a cellular scar tissue.^34^ These data suggest that soft plaques seen in young patients may have been present for a shorter time of period than plaques in older patients, which have a large content of fibrous tissue and are responsible for most episodes of major coronary thrombosis.^35^ The less extensive CAD observed in younger patients in our study might suggest that premature CAD is associated with short-lived and rapid disease progression rather than with a gradually evolving process. This is in agreement with the previous observations that, STEMI is the common first presentation in younger patients.^32^–^33^

In agreement with our results, Borrás Pallé et al. 36 analyzed the inflammatory state of 61 patients with UA and NSTEMI using Hs-CRP, leukocytes and fibrinogen as inflammatory markers. They found that, despite elevated levels of Hs-CRP in ACS groups these markers were not related to the risk of cardiovascular complications.

In contrast Correia et al.^37^ studied CRP measured at admission of patients with non-ST-elevation ACS and concluded that CRP adds prognostic information to the TIMI-Risk Score and the incorporation of this variable into the TIMI-Risk Score calculation is a useful ingredient.

## Precedent Outlook

Several trials have evaluated the predictive value of CRP and Hs-CRP for cardiovascular risk. However, much remains to be learned to explain CRP’s association with risk. Levels of CRP that define increased risk vary depending on the population studied. The degree of elevation of CRP that identifies increased risk has not been clearly determined, and the clinical utility of CRP after adjustment for angiographic findings has been uncertain. C-reactive protein previously has been proposed to correlate with the extent of atherosclerosis.^38^,^39^ Studies have shown that lower profile plaques are more numerous than “significant stenoses” and therefore, are statistically more likely to lead to plaque rupture than the relatively few lesions of ≥70% stenosis.^40^ Thus, it is possible that an elevated CRP simply represents a more diffuse process of coronary atherosclerosis with a higher total plaque burden. If so, the predictive value of CRP would be considerably less after adjustment for extent of disease (total number of lesions) assessed by coronary angiography. To exclude this possibility, the predictive value of CRP must be adjusted for measures of all visible atherosclerotic plaques on angiography. On the other hand, if CRP levels primarily represent plaque properties (i.e. inflammation, instability), knowledge of CRP levels would continue to be useful even in the presence of angiographic assessment of plaque burden (extent/severity). Similarly, CRP and coronary calcium score (assessed by ultrafast computed tomography) may have independent and additive prognostic value, but this has not been extensively evaluated.

In the present study a negative correlation was found between Hs-CRP levels and ejection fraction while Hs-CRP levels where positively correlated with angiography findings (number of stenotic vessels, lesion scoring) only in older study groups. Again regarding the mechanisms implicated on the prediction of coronary disease severity by Hs-CRP, it can be related to either a direct or indirect effect of this protein. During ACS a considerable increase in plasma CRP takes place as part of the acute phase reaction.^41^

In our study, ACS younger patients (with less extensive CAD), Hs-CRP was not a good predictor of atherosclerotic severity. In these circumstances, the high concentrations of Hs-CPR may be not a marker of chronic atherosclerotic changes with acute exacerbation but rather a result of acute plaque rupture. So Hs-CRP could be used as a risk predictor for acute cardiovascular events. It is documented that CRP activates complement which may cause myocardial damage, predisposing to heart failure or arrhythmias especially following acute vascular stress.^42^ Lastly the increase in plasma Hs-CRP levels in young during ACS seems to be linked to pre-existing inflammation rather than the extension of myocardial damage.

## Study Limitations

Quantification of coronary angiographic findings was limited to the visual interpretation of the attending cardiologist, which is representative of “real-world” practice. Intravascular ultrasound could be expected to give increased measures of atherosclerotic burden by identifying “intramural plaques,” although this would be impractical to apply routinely. However, whether intravascular ultrasound assessments would adversely impact predictive value of CRP is unclear.

Only patients with clinical presentations suggesting unstable angina or infarction who underwent angiography were included in our study. However, the vast majority of patients undergoing angiography today do so for evaluation of suspected angina. Many of these patients (about 20%) have normal angiograms. It is often difficult to determine which of these patients have true angina related to occult microvascular disease or endothelial dysfunction and which truly have non-cardiac chest discomfort. Hs-CRP thus appears to be useful in evaluating risk in patients without visible CAD, although the beneficial effects of more aggressive medical therapy in these patients^43^,^44^ should be further evaluated.

## Conclusion

Our study shows a significantly different clinical, angiographic and biochemical profile in young patients (<45 years) with ACS compared with those ≥45 years ones. Patients with premature CAD commonly have different risk profile, less extensive CAD than the older ones.

Hs-CRP plasma concentrations showed a different release pattern in young patients with ACS in comparison with elderly. Hs-CRP peak concentrations did not correlate with angiographic findings in young that could be attributed to different risk profile.

In our study and consistent with epidemiologic studies; positive family history, smoking, elevated triglycerides levels and low HDL-cholesterol level were also associated with premature ACS. Dominance of smoking and dyslipidemia that are the preventable risk factors in premature CAD patients is an important threat for young adult. Healthy life styles should be encouraged beginning from young ages and new precautions about smoking must be taken.

## Figures and Tables

**Table 1.** Risk profile of study patients with ACS.

	**Group I (n = 68)**	**Group II (n = 110)**	**Group III (n = 75)**
Age in years (mean ±SD)	33.2 ± 2.9	50.2 ± 4.8*	69.3 ± 3.5**
Male	64 (94.1%)	70 (72.7%)*	60 (80%)*
Female	4 (5.9%)	40 (27.3%)*	15 (20%)*
BMI > 25 kg/m^2^ (%)	32 (47%)	74 (67%)*	50 (67%)*
Smoking (%)	54 (79%)	40 (36%)*	32 (42%)*
Hypertension (%)	15 (23%)	55 (59%)*	49 (65%)*
Diabetes mellitus (%)	5 (7%)	33 (30%)*	40 (53%)**^
Positive FH (%)	30 (44%)	22 (20%)*	10 (13%)*
Hypercholesterolemia (%)	26 (38%)	50 (45%)*	35 (47%)*
Hypertriglyceredemia (%)	36 (53%)	40 (36%)*	30 (40%)*
Low HDL (%)	52 (76%)	51 (46%)*	45 (60%)*^

**Abbreviations:** BMI, body mass index; UA, unstable angina; NSTEMI, non ST elevation myocardial infarction; STEMI, ST elevation myocardial infarction; ACEI, angiotensin converting enzyme inhibitor.

* gp II and III vs. I, P < 0.01.

** gp II and III vs. I, P < 0.001.

^ grp III vs. II, P < 0.01.

**Table 2:** Comparison of laboratory findings between studied groups.

	**Group I (n = 68)**	**Group II (n = 110)**	**Group III (n = 75)**
Fasting glucose, mg/dl	92 ± 15	127 ± 24*	146 ± 27**^
Total cholesterol, mg/dl	192 ± 25	233 ± 43**	230 ± 39**
LDL-cholesterol, mg/dl	120 ± 12	147 ± 28**	140 ± 28*
HDL-cholesterol, mg/dl	34 ± 13	43 ± 15*	42 ± 18*
Triglyceride, mg/dl	158 ± 27	140 ± 14**	145 ± 17*
CPK	534.9 ± 423.3	743 ± 345.5*	814.6 ± 431.7*
CK-Mb	28.3 ± 23.4	34.8 ± 26.7*	32.9 ± 37.3*
LDH	645 ± 158.4	823.7 ± 650*	798.9 ± 643.3*
Hs-CRP (mg/l)	6.1 ± 1.2	8.4 ± 2.9*	11.5 ± 3.2**^
Serum creatinine (μg/l)	0.76 ± 0.21	0.82 ± 0.34	0.96 ± 0.67*
Blood Urea (mmol/l)	29 ± 3.4	31 ± 4.9	34 ± 7.9*

**Abbreviations:** CPK, creatinine phosphokinase; LDH, lactic dehydrogenase; Hs-CRP, high sensitive C-reactive protein.

* gp II and III vs. I, P < 0.01.

** gp II and III vs. I, P < 0.001.

^ grp III vs. II, P < 0.01.

**Table 3:** Angiographic data in the different study groups.

	**Group I (n = 68)**	**Group II (n = 110)**	**Group III (n = 75)**
**Vessel score**			
0	3 (4%)	0	0
1	45 (66%)	42 (38%)**	20 (27%)**^
2	14 (21%)	39 (35%)**	25 (33%)**
3	6 (9%)	29 (27%)**	30 (40%)**^
**Involvement of coronary arteries**			
Left main	1 (1%)	5 (5%)	3 (4%)
LAD	48 (71%)	71 (64.5%)*	69 (92%)*^
LCX	9 (13%)	61 (55%)**	27 (36%)*^
RCA	16 (24%)	55 (50%)**	18 (24%)^
**Coronary Score**	4.77 ± 2.50	6.49 ± 6.17*	9.43 ± 6.61**^

**Abbreviations:** LAD, left anterior descending; LCX, left circumflex; RCA, right coronary artery.

* gp II and III vs. I, P < 0.01.

** gp II and III vs. I, P < 0.001.

^ grp III vs. II, P < 0.01.

**Table 4:** Relationship of Hs-CRP and other clinical variables to the severity of coronary diseases in different study groups.

**Coronary severity score**	**Group I (n = 68)**	**Group II (n = 110)**	**Group III (n = 75)**
Hs-CRP	r 0.051	r 0.239	r 0.334
	p 0.369	p 0.034	p 0.012
Age	r 0.356	r 0.363	r 0.434
	p 0.011	p 0.024	p 0.001
Gender (male)	r 0.401	r 0.321	r 0.255
	p 0.012	p 0.031	p 0.041
BMI > 25 Kg/m^2^	r 0.151	r 0.021	r 0.074
	p 0.461	p 0.829	p 0.669
Smoking	r 0.450	r 0.162	r 0.251
	p 0.001	p 0.481	p 0.363
Hypertension	r 0.001	r 0.026	r 0.011
	p 0.532	p 0.569	p 0.769
Diabetes mellitus	r 0.032	r 0.551	r 0.451
	p 0.469	p 0.001	p 0.009
Total cholesterol	r 0.150	r 0.247	r 0.198
	p 0.389	p 0.089	p 0.095
Triglyceride	r 0.201	r 0.071	r 0.078
	p 0.051	p 0.429	p 0.521
LDL	r 0.032	r 0.155	r 0.064
	p 0.460	p 0.476	p 0.257
HDL	r 0.211	r 0.091	r 0.051
	p 0.071	p 0.466	p 0.268
CPK	r 0.651	r 0.451	r 0.551
	p 0.019	p 0.013	p 0.039
LDH	r 0.591	r 0.511	r 0.498
	p 0.009	p 0.002	p 0.012
EF%	r −0.411	r −0.311	r −0.453
	p 0.019	p 0.029	p 0.012

**Abbreviations:** Hs-CRP, high sensitive; C-reactive protein. BMI, body mass index; LDL-C, low density lipoprotein; HDL-C, high density lipoprotein; CPK, creatinine phosphokinase; LDH, lactic dehydrogenase; EF%, ejection fraction.

**Table 5:** Multivariate predictors of the extent of CAD in the young patients (group I).

	**Odd ratio (OR)**	**95% Confidence interval (CI)**	**T**	**P value**
Age	1.194	1.045–1.363	2.42	0.009
Gender (male)	1.235	1.046–2.123	3.57	0.007
Smoking	0.121	0.023–0.226	2.05	0.0001
Triglycerides	0.001	1.05–2.33	0.065	>0.05
HDL-C	1.176	0.0934–2.143	2.65	<0.001
CPK	0.052	0.031–0.173	2.54	<0.001
LDH	0.182	0.11–0.360	3.38	<0.001
EF%	0.341	0.105–2.36	2.14	<0.01

**Abbreviations:** HDL-C, high density lipoprotein; CPK, creatinine kinase, LDH, lactic dehydrogenase, EF%, ejection fraction.

**Table 6:** Multivariate predictors of the extent of CAD in the older patients with ACS (group II and III).

	**Odd ratio (OR)**	**95% Confidence interval (CI)**	**T**	**P value**
Age	1.120	1.045–1.214	2.42	<0.003
Gender (male)	0.005	1.016–1.523	0.057	>0.05
Diabetes mellitus	0.167	0.124–0.326	3.05	<0.0001
Hs-CRP	2.01	1.14–3.23	4.065	<0.007
CPK	0.042	0.022–1.153	2.58	<0.001
LDH	0.172	0.141–0.260	2.38	<0.001
EF%	1.342	0.873–2.300	3.121	<0.001

**Abbreviations:** Hs-CRP, high sensitive C-reactive protein; CPK, creatinine kinase; LDH, lactic dehydrogenase; EF%, ejection fraction.
